# The BrainHealth Databank: a systems approach to data-driven mental health care and research

**DOI:** 10.3389/fninf.2025.1616981

**Published:** 2025-08-13

**Authors:** Jose Arturo Santisteban, David Rotenberg, Stefan Kloiber, Marta M. Maslej, Adeel Ansari, Bahar Amani, Darren Courtney, Farhat Farrokhi, Natalie Freeman, Masooma Hassan, Lucia Kwan, Mindaugas Mozuraitis, Michael Lau, Natalia Potapova, Farhad Qureshi, Nicole Schoer, Nelson Shen, Joanna Yu, Noelle Coombe, Kimberly Hunter, Peter Selby, Nicole Thomson, Damian Jankowicz, Sean L. Hill

**Affiliations:** ^1^Krembil Centre for Neuroinformatics, Centre for Addiction and Mental Health, Toronto, ON, Canada; ^2^Centre for Addiction and Mental Health, Campbell Family Mental Health Research Institute, Toronto, ON, Canada; ^3^Department of Psychiatry, University of Toronto, Toronto, ON, Canada; ^4^Cundill Centre for Child and Youth Depression, Centre for Addiction and Mental Health, Toronto, ON, Canada; ^5^Data and Insights, Information Management Group, Centre for Addiction and Mental Health, Toronto, ON, Canada; ^6^Centre for Addiction and Mental Health, Toronto, ON, Canada; ^7^Safehaven, Toronto, ON, Canada; ^8^Institute of Health Policy, Management and Evaluation, University of Toronto, Toronto, ON, Canada; ^9^Waterloo Regional Health Network, Kitchener, ON, Canada

**Keywords:** learning health system, system approach, mental health, data, Open Science, AI, digitization

## Abstract

**Introduction:**

Mental health care is undermined by fragmented data collection, as incomplete datasets can compromise treatment efficacy and research. The BrainHealth Databank (BHDB) at the Centre for Addiction and Mental Health (CAMH) establishes the governance and infrastructure for a Learning Mental Health System that integrates digital tools, measurement-based care, artificial intelligence (AI), and open science to deliver personalized, data-driven care.

**Methods:**

Central to the BHDB’s approach is its comprehensive governance framework, which actively engages clinicians, researchers, data scientists, privacy and ethics experts, and patient and family partners. This codesigned approach ensures that digital health technologies are deployed ethically, securely, and effectively within clinical settings.

**Results:**

By aligning data collection with clinical and research goals and harmonizing over 12 million data points from 33,000 patient trajectories, the BHDB enhances data quality, enables real-time decision support, and fosters continuous improvement.

**Discussion:**

The BHDB provides a model for integrating AI and digital tools into mental health care, as well as research data collection, analyses, storage, and sharing through the BHDB Portal (https://bhdb.camh.ca).

## Introduction

Mental health disorders represent a significant global health challenge (Üstün and Kennedy, 2009), contributing to 32.4% of years lived with disability worldwide (Vigo et al., 2016). The economic burden is equally profound, with mental illness in Canada alone costing an estimated $51 billion in 2003 (Lim et al., 2008). Despite the urgent need for more effective mental health care systems, the field continues to face substantial challenges, including fragmented data collection, inconsistent application of evidence-based practices, and limited patient engagement (Dewa et al., 2011; Guo et al., 2015; Fava and Davidson, 1996; De Carlo et al., 2016; Kolovos et al., 2017; Dold and Kasper, 2017). These issues lead to incomplete datasets, which undermines the quality of care and hinders the progress of mental health research (Lam et al., 2016).

Measurement-based care (MBC), which involves the systematic evaluation of patient symptoms to inform treatment, has been recognized as a crucial component in addressing these challenges (Lewis et al., 2019). MBC empowers clinicians with quantitative data to assess treatment responses, modify interventions, and compare outcomes with those in the scientific literature (Howard et al., 1996). This approach enhances clinical outcomes by enabling rapid detection of deteriorating symptoms and effectively managing persistent conditions (Lewis et al., 2019). However, despite its clear benefits (Guo et al., 2015; Lewis et al., 2019), MBC remains an underutilized approach in mental health care, with fewer than 20% of practitioners regularly applying it in practice (Lewis et al., 2019; Zimmerman and McGlinchey, 2008). This underutilization is often due to inconsistent use of standardized assessment tools, resulting in fragmented and incomplete data collection (Barch et al., 2016).

To address these challenges, the Centre for Addiction and Mental Health (CAMH), Canada’s largest mental health hospital, launched the BHDB initiative. The BHDB aims to establish a Learning Mental Health System (LMHS) to integrate advanced digital tools, MBC, research methodologies, artificial intelligence (AI), and open science (Smith et al., 2013). This initiative is particularly relevant in the evolving landscape of AI and digital health technologies, where the need for robust governance models to ensure the ethical, secure, and effective implementation of these technologies is increasingly critical (Guidance, 2021).

A cornerstone of the BHDB is its comprehensive governance framework, which is designed to navigate the complex ethical, legal, and practical challenges associated with integrating AI and digital health technologies into mental health care. However, a key motivation behind this governance model is to ensure that the data collected are of mutual value to all partners and meet the highest standards of quality. By actively engaging a diverse array of partners—including clinicians, researchers, privacy and ethics experts, patient and family representatives, and data scientists—the BHDB ensures that the system operates transparently, ethically, and effectively. Such inclusive governance is crucial for building trust, ensuring accountability, and fostering a collaborative culture where all partners find value in the data, supporting continuous learning and improvement in mental health care.

The governance framework is more than a structural component. The framework is a dynamic process that facilitates the alignment of the BHDB with ethical standards, legal requirements, and best practices in data management. It also supports continuous monitoring and evaluation, ensuring that the system remains responsive to emerging challenges and opportunities. By involving all relevant partners in decision-making, the BHDB governance model ensures that the data collected are not only high quality but also relevant and actionable, directly contributing to better patient outcomes and more effective research.

Data-driven precision medicine and evidence-based mental healthcare have long been proposed as solutions for improving mental health outcomes (Howard et al., 1996; Donabedian, 1980). However, the application of these approaches has been limited by a lack of scalable strategies and the digital tools necessary to implement them effectively (Li et al., 2021). This challenge is particularly pronounced in mental health care, where the heterogeneity of psychiatric disorders and the reliance on self-report measures complicate the standardization and integration of data (Barch et al., 2016). The BHDB addresses these challenges by leveraging digital sources, such as electronic health records (EHR) and wearable technologies, to enhance data-driven approaches and standardize data collection across the mental health landscape.

Moreover, the BHDB’s systems approach is particularly vital in the context of mental health care, where the complexity and variability of conditions demand adaptive and responsive solutions (Pei et al., 2021). By harmonizing and integrating multidimensional data across clinical and research domains (Rotenberg et al., 2018), the BHDB enables real-time decision-making, personalized treatment strategies, and ongoing innovation. This approach not only enhances the quality of care delivered at CAMH but also provides a scalable model that can be adapted to other healthcare institutions, extending the impact of AI and digital health technologies across the broader healthcare landscape.

Central to the BHDB’s success is the principle of codesign (Sanz et al., 2021), where partners are not only consulted but also actively involved in the development of digital tools and processes (Yu et al., 2023). This ensures that the tools are user friendly, relevant, and aligned with clinical needs, fostering trust, accountability, and a collaborative culture that supports continuous learning and improvement. The success of the BHDB at CAMH will be assessed through several key metrics, including improvements in data quality and integration, enhanced clinical outcomes driven by AI-supported decision-making, and increased partner engagement and trust in the governance process.

As the BHDB continues to evolve, its outcomes will offer valuable insights into how AI-driven systems, supported by MBC, codesigned, and robust governance models, can help shape the landscape of mental health care globally. This initiative represents a significant advancement in the integration of AI and digital health technologies within mental health care, providing a model for how these technologies can be ethically and effectively deployed in complex healthcare environments.

## Methods

### Governance committees and working groups

**Figure 1:**
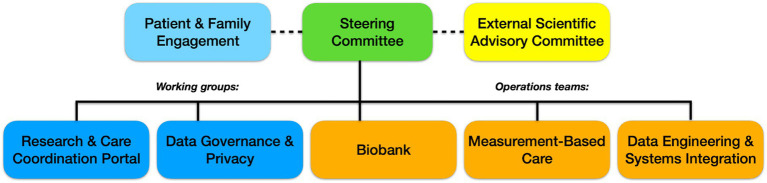
The governance structure of the BrainHealth Databank (BHDB) includes a steering committee composed of senior leaders, clinical directors, and research heads overseeing the initiative. The External Scientific Advisory Committee consists of external experts in AI, mental health, ethics, and open science, providing strategic guidance. Various working groups include professionals such as clinical researchers, IT specialists, data managers, and patient advocates, who focus on areas such as the Research & Care Coordination Portal, Data Governance & Privacy, and Patient & Family Engagement. The operations teams are composed of laboratory scientists, software engineers, and clinical care providers, managing key areas such as the biobank, data engineering and systems integration, and measurement-based care (MBC) to ensure the smooth implementation of BHDB’s objectives.

Effective governance has been integral to the success of the BHDB initiative. The governance structures (Figure 1) were designed to ensure the active involvement of all the partners, which is crucial for maintaining trust, transparency, and accountability throughout the project. Committees provide higher-level strategic direction for the BHDB, either through leadership (in the case of the Steering Committee) or by providing recommendations (in the case of the External Scientific Advisory Committee). The Working Groups implement the strategic goals determined by the Steering Committee. These structures align the initiative with ethical standards, legal requirements, and best practices in data management. Additionally, they support continuous monitoring and evaluation, allowing the BHDB to adapt and evolve in response to feedback and changing needs.

*Metrics.* The number of partners was obtained from membership lists from the committees’ terms of reference. Partner feedback was recorded during meetings as actionable items to determine if they led to changes. Patient and family partner satisfaction were obtained by administering the Public and Patient Engagement Evaluation Tool (PPEET), a questionnaire to evaluate public and patient engagement developed primarily for use within health system organizations (Abelson et al., 2016). Number of organizations working with CAMH to implement BHDB infrastructure. Number of citations for the use of BHDB governance based on a literature search of BHDB governance publications and study protocols that use BHDB tools.

### Governance committees

1. Steering Committee: The steering committee serves as the primary decision-making body for the BHDB. It includes representatives from key clinical and research departments, the privacy office, the patient and family engagement team, and other partners, such as the director of the Krembil Centre for Neuroinformatics (KCNI). The committee’s main responsibility is to ensure that the BHDB remains aligned with CAMH’s strategic goals. The Steering Committee meets bimonthly to review progress, address challenges, and make strategic decisions. It reports directly to the Discovery Fund Steering Committee (not shown), ensuring that all activities align with institutional objectives and governance requirements.
2. External Scientific Advisory Committee: This committee consists of leading international experts in LHS and open science. They provide strategic advice, recommend best practices, and ensure that the BHDB aligns with global data initiatives and standards. The committee meets quarterly and provides feedback on the direction of the BHDB to maintain alignment with cutting-edge research and global best practices.

### Working groups

1. Data Governance and Privacy Working Group: This group oversees the lawful, secure, and ethical handling of all BHDB data. The group is responsible for creating and updating policies that govern data collection, use, storage, and disposal, ensuring compliance with privacy regulations such as the United States’ Health Insurance Portability and Accountability Act (HIPAA), the European Union’s General Data Protection (GDPR), Canada’s Personal Information Protection and Electronic Documents Act (PIPEDA), and Ontario’s Personal Health Information Protection Act (PHIPA). The working group has also developed a comprehensive data governance framework that includes regular audits, risk assessments, and deidentification protocols to protect patient privacy.
2. Patient and Family Engagement Team: Comprising patient advisors and family representatives, this team ensures that the BHDB reflects the needs and values of the people it serves. The team is actively involved in the codesign of tools and workflows, providing critical input on how the system can better meet patient and caregiver needs. Their involvement extends to participation in the Steering Committee, where they help shape the strategic direction of the BHDB.
3. Biobank Working Group: This group manages the collection, storage, and use of biological samples associated with the BHDB. The group ensures that CAMH Biobank and Molecular Core Facility operations align with clinical research processes and support open science principles (Poupon et al., 2017). Policies regarding the secondary use of biological research samples are regularly reviewed and updated to facilitate research while protecting participant rights.
4. Research and Care Coordination Portal Working Group: This group was instrumental in developing the MyCAMH Portal, which provides a centralized platform for data access and collaboration among researchers and clinicians. The working group gathered requirements from various partners to ensure that the portal met the needs of both the research and the clinical teams. The portal was launched in February 2024 and continues to be refined on the basis of user feedback.
5. Data Engineering and Systems Integration Working Group: This technical team is responsible for maintaining the technical components of the BHDB. This includes integrating various data sources through research and clinical knowledge graphs, maintaining the data warehouses and lakes, maintaining high standards of data quality, and ensuring real-time data processing, as well as helping users with data analytics (i.e., using Python and R for analyses and Tableau, and Apache Superset for visualization). The group developed the BHDB Knowledge Graph using the Blue Brain Nexus (Sy et al., 2023) and implemented the fast healthcare interoperability resources (FHIR) standard (Bender and Sartipi, 2013) to enable seamless data interoperability. Typical tasks for the data engineering team include data ingestion from systems, such as the electronic medical record, REDCap, XNAT, Labkey, and wearable devices; data integration and standardization according to HL7 FHIR, LOINC and SNOMED, and data mart creation for specific clinical and research teams. The team’s work ensures that all the data entering the BHDB are standardized, auditable, and reusable.
6. Measurement-Based Care (MBC) Working Group: Initially, focused on the MDD-ICP, this group now oversees the standardization of measurement tools across different care pathways and clinics. The group’s role is to ensure that common data elements and technologies are consistently applied, facilitating data comparison and continuous quality improvement. This working group has evolved into the MBC User Group, which provides ongoing feedback and suggestions for enhancing digital care pathways.

### Team required for implementation

The successful implementation and ongoing operation of the BHDB required a multidisciplinary team with specialized expertise across various domains:

- Data Scientists: This team was responsible for developing and maintaining the BHDB knowledge graph (Rotenberg et al., 2018; Sy et al., 2023). They ensured that data integration was seamless by applying the FHIR standard (Bender and Sartipi, 2013) to harmonize data from different sources. Data scientists also collaborated with clinicians to ensure that the data models were aligned with clinical workflows, enabling accurate and relevant data analysis.
- Clinicians and researchers: Clinicians and researchers play critical roles in mapping MBC pathways (Hawley et al., 2021). They provided the clinical insights necessary to guide the development of decision support tools, ensuring that these tools were clinically relevant and could be easily integrated into daily practice. Their contributions were essential to the ability of the BHDB to meet the needs of both clinical care and research.
- Privacy and ethics experts: This team ensured that the BHDB complied with all relevant privacy regulations, including the HIPA, GDPR, PIPEDA, and PHIPA. They developed robust data governance measures, including deidentification processes, to protect patient privacy while allowing meaningful data use. They were also involved in crafting the policies and procedures that govern the ethical use of data within the BHDB.
- Patient and Family Representatives: Active participants in the codesign of digital tools and patient and family representatives provided ongoing feedback to ensure that the BHDB met the needs of patients and caregivers. Their input was critical in creating tools that were user friendly, relevant, and trusted by the patient community.
- Technical and IT Support Staff: This team managed the technical infrastructure of the BHDB, including the deployment of the Blue Brain Nexus (Sy et al., 2023), the integration of digital tools such as REDCap (Rotenberg et al., 2018; Hawley et al., 2021), and the maintenance of the overall system. They ensured that the technology supporting the BHDB was reliable, scalable, and secure, providing the necessary backbone for all data processing and analysis activities.
- Governance and administrative staff: These staff members supported the governance processes, coordinated partner engagement, and ensured that the BHDB’s operations were aligned with institutional policies. Their work was crucial in maintaining the organizational structure and ensuring that all governance activities were conducted efficiently and transparently.

### Infrastructure for a learning health system (LHS)

The BHDB provides the essential infrastructure necessary to establish and maintain an LMHS. Leveraging advanced digital tools, robust IT infrastructure, and a comprehensive data integration framework, the BHDB supports continuous data collection (Hawley et al., 2021), analysis, and application of multidimensional data (Rotenberg et al., 2018). The system’s core deliverables align with the principles of LHS, emphasizing continuous learning, real-time data feedback, and a culture of improvement. The key components of the BHDB infrastructure include.

### Digital support for measurement-based care (MBC)

- Digitization of Clinical Care Pathways: The BHDB integrates digital tools to enable the systematic evaluation of patient symptoms, facilitating MBC. This includes standardized assessment tools, EHRs, and digital data collection platforms such as REDCap (Hawley et al., 2021). These tools ensure consistent data collection that is readily available for clinical use and research purposes. New measures are reviewed by the CAMH Standards and Measures Committee to ensure that the measures are aligned across pathways. These measures are available on the Neuroinformatics Platform upon request to clinicians, hospital administrations, and researchers (with ethical approval) (Rotenberg et al., 2018).

*Metrics.* The number of pathways, clinicians, data points, standardized questionnaires, and patient trajectories were derived from counts of the data collected through REDCap. Improvement in completeness was determined from comparing the number of complete records when the form was completed through a clinical interview versus after the implementation of a self-administered sociodemographic form on REDCap.

- Digital Dashboards and Visualization Tools: The BHDB provides digital dashboards and visualization tools that facilitate real-time data capture and presentation. These tools, which are codesigned with clinicians, integrate seamlessly into clinical workflows, providing timely and actionable insights that support decision-making (Hassan et al., 2024b).

*Metrics.* The impact of implementing the dashboard was obtained by comparing the mean improvement from the initial appointment to the final appointment of patients before the dashboard was implemented versus after the dashboard was implemented and in use. Clinician engagement was derived from REDCap data on the number of clinician users and the total number of CAMH physicians.

### Integration of research measures into care pathways

- Tailored protocols: The BHDB enables the direct integration of research measures into clinical care pathways through tailored protocols. This ensures that data collected during routine care can be used for research without disrupting clinical workflows. Standardized assessment tools and protocols are applied across all pathways to ensure data consistency and reliability.
- *Metrics.* Number of studies and participants in studies that utilize data from the BHDB. Number of publications derived from a literature search of peer-reviewed publications and conference proceedings, as well as a survey of research staff.
- Knowledge Graph: The BHDB knowledge graph, developed via the Blue Brain Nexus (Sy et al., 2023), integrates data from various sources, such as EHRs, biobanks, and wearable devices (Rotenberg et al., 2018). The knowledge graph employs the HL7 FHIR standard (Bender and Sartipi, 2013) to ensure data interoperability and accessibility across different platforms, enabling a comprehensive view of patient data for both clinical care and research.

*Metrics.* Laboratory data were obtained from the CAMH Biobank and Molecular Core records within LabKey.

### AI and data-driven personalized care

- Machine Learning Models: Future applications of the BHDB will further leverage AI and machine learning to analyze clinical data and generate actionable insights. These models are being trained on large and diverse BHDB datasets to identify patterns, predict outcomes, and inform personalized treatment plans, with the aim of improving patient care.
- Clinical decision support: AI-driven decision support tools utilize data from the knowledge graph to provide clinicians with real-time, data-driven insights. These tools enhance clinical decision-making by integrating a wide range of patient data into the decision-making process, supporting personalized and precision medicine.

### Open science for discovery and innovation

- Data Sharing Policies: The BHDB promotes open science principles (Poupon et al., 2017) by providing the infrastructure necessary for secondary data reuse. This approach supports transparency, collaboration, and innovation in mental health research, ensuring that the data collected contribute to broader scientific discovery.
- Research (biological) sample sharing policies: Samples collected from participants who have provided explicit consent to share their samples will be managed and stored in the CAMH Biobank and Molecular Core Facility. This facility has processed and stored biological samples for research studies for more than 20 years at CAMH and has validated processes and procedures for managing BHDB samples in place. Research studies wishing to use BHDB samples for secondary use require approval by the CAMH Research Ethics Board and the BHDB Biobank Working Group prior to commencing the study.

### Digital support for measurement-based care (MBC)

The BHDB provides a comprehensive suite of tools to support MBC, facilitating the systematic collection, integration, and analysis of multidimensional data (Hawley et al., 2021). This approach ensures high data quality, promotes interoperability, and engages all partners in the data collection and integration process.

Foundation of Care Pathways: The BHDB was built on the foundation of care pathways developed by CAMH, which are multidisciplinary structured care plans detailing essential, sequential steps for patient care. Implementing these pathways ensures that MBC is systematically integrated into clinical workflows (Hawley et al., 2021).

Deployment of REDCap: CAMH deployed a clinical instance of REDCap, a secure web application for managing electronic surveys and patient self-assessments, across CAMH clinics and departments. REDCap automatically transfers data to the EHR in both discrete and PDF formats, streamlining data collection and integration. Patients receive a personalized link through email before their appointments and then complete self-assessment surveys digitally via standardized measurements (Hawley et al., 2021).

### Ensuring data quality and interoperability

Data quality is critical to the success of the BHDB and is assessed through several specific methodologies:

1. Defining data quality

Engaging with partners: The BHDB engaged with partners to define the measures of quality, metrics, and acceptability thresholds for these measures.

2. Data accuracy

Validation Checks: Automated validation rules within REDCap and other data entry systems ensure that the data entered meet predefined criteria such as correct formats, ranges, and logical consistency. Cross-verification of key data points against reliable sources such as EHRs further enhances accuracy (Hawley et al., 2021).

3. Data completeness

Missing Data Analysis: Regular audits identify and address missing data, with real-time feedback provided to clinicians. The completeness of the data is continuously monitored, and corrective actions are taken when thresholds for missing data are exceeded.

4. Data consistency

Standardization protocols: Consistency is maintained by using standardized assessment tools and questionnaires across all care pathways (Hawley et al., 2021). Data harmonization processes ensure uniformity across different data sources by mapping data to the FHIR standard (Bender and Sartipi, 2013).

5. Data timeliness

Real-time data capture: Digital tools enable real-time data entry, ensuring that the data are current and available for immediate use (Rotenberg et al., 2018). Timeliness metrics, such as the average time from data entry to availability in the knowledge graph, are tracked and optimized.

6. Error detection and correction

Data Cleaning Procedures: Automated scripts in Python identify and correct duplicate entries, outliers, and inconsistencies. Manual reviews by data scientists and clinicians further ensure data integrity.

7. Data integrity

Audit Trails and Version Control: The BHDB maintains audit trials for all data entries and modifications (Rotenberg et al., 2018). Version control allows tracking of changes over time, ensuring transparency and data integrity.

8. User training and support

Training programs: Clinicians, researchers, and data entry personnel receive training on data entry protocols and the importance of data quality. Ongoing support is provided through help desks and refresher sessions.

9. Continuous monitoring and improvement

Quality Dashboards: Real-time dashboards monitor key data quality indicators, providing visibility and highlighting areas requiring intervention. Targeted quality improvement initiatives are launched on the basis of these insights.

### Integration of research into care pathways

The BHDB facilitates the seamless integration of research measures into existing care pathways, bridging clinical practice and research to enhance the quality and effectiveness of both. This integration ensures that data collected during routine care can be utilized for research purposes, creating a robust framework that supports continuous learning and improvement.

Facilitating Integration of Research Measures: The BHDB achieves the integration of research measures into clinical care pathways through several key strategies. Tailored protocols are developed to incorporate research measures into digitized care pathways, ensuring that data collected during routine clinical care are systematically captured for research purposes without disrupting clinical workflows. Standardized assessment tools and data collection protocols are used across care pathways to ensure consistency and reliability (Hawley et al., 2021).

Systems Approach to Integration: A systems approach is essential to ensure the seamless integration of research measures into existing care pathways (Clarkson et al., 2018). This involves engaging a wide range of partners, including clinicians, researchers, privacy and ethics experts, patient and family representatives, and data scientists, to ensure that the integration process is comprehensive and considers multiple perspectives. Continuous feedback mechanisms are built into the system to ensure that research measures are effectively integrated and provide value to clinical care, allowing the system to adapt and evolve on the basis of real-world evidence and partner input.

### Artificial intelligence and data-driven personalized care

The BHDB leverages clinical data, advanced analytics, and machine learning to support personalized care. This innovative approach, currently under development, has the potential to predict responses to mental health treatments, benefiting clinicians, patients, and family members.

Using Clinical Data and Machine Learning: The BHDB utilizes a wealth of clinical data collected from EHRs, digital assessments, wearable devices, and other sources. These data are processed and analyzed via advanced analytics and machine learning algorithms to generate actionable insights. Machine learning models are being developed and trained on large datasets from clinical data collected during the course of treatment to identify patterns and predict outcomes, enabling clinicians to make data-driven decisions tailored to individual patient needs. The KCNI is developing the Brain Health Data Challenge platform, where large datasets derived from the BHDB and other Open Science datasets will be analyzed with machine learning and AI methods to solve specific challenges, such as psychosis risk prediction, digital mental health service access and quality of care, and substance use disorder treatment response prediction.

Advanced Analytics: The BHDB employs advanced analytics to process and analyze vast amounts of clinical data. This includes statistical analysis, predictive modeling, and pattern recognition, which help in understanding patient behaviors, treatment responses, and potential outcomes.

### Open science for discovery and innovation

Open science plays a crucial role in accelerating research and discovery in mental health by promoting transparency, collaboration, and accessibility (Vicente-Saez and Martinez-Fuentes, 2018). Sharing data and resources reduces redundancy, increases resource efficiency, and improves transparency, thereby enhancing public trust and support for mental health research. The BHDB embodies these principles (Poupon et al., 2017), providing an extensive digital repository of mental health data that enhances research capabilities. In addition to the CAMH Biobank and Molecular Core, the BHDB supports the sharing and use of biological samples and associated digital research data. This commitment to ethical open science fosters trust and collaboration among partners, accelerates research and discovery, and ensures that the benefits of research are widely accessible. Through its systematic approach to data management and sharing, the BHDB drives innovation in mental health research, ultimately improving outcomes for patients worldwide.

*Comparison with related data repositories for secondary research:* An environmental scan of data repositories providing access to data for secondary use in research was performed. This environmental scan was performed with the aim of capturing the wide, but not exhaustive, range of health data repositories, with a focus on mental health and neuroscience data repositories. This scan was done by searching on internet search engines in two ways: (a) directly searching for repositories that were known by the BHDB Team and (b) searching for combinations of ‘neuro’, ‘mental health’, ‘psychiatry’, ‘genetic’, ‘health’, ‘medicine’, and ‘medical’ with ‘data’, ‘repository’, ‘secondary’, ‘re-use’, and ‘sharing’. The inclusion criteria were (a) data repository that collects human health data and (b) the data is shared for unspecified future re-use. There were no exclusion criteria.

### Data flow

**Figure 2:**
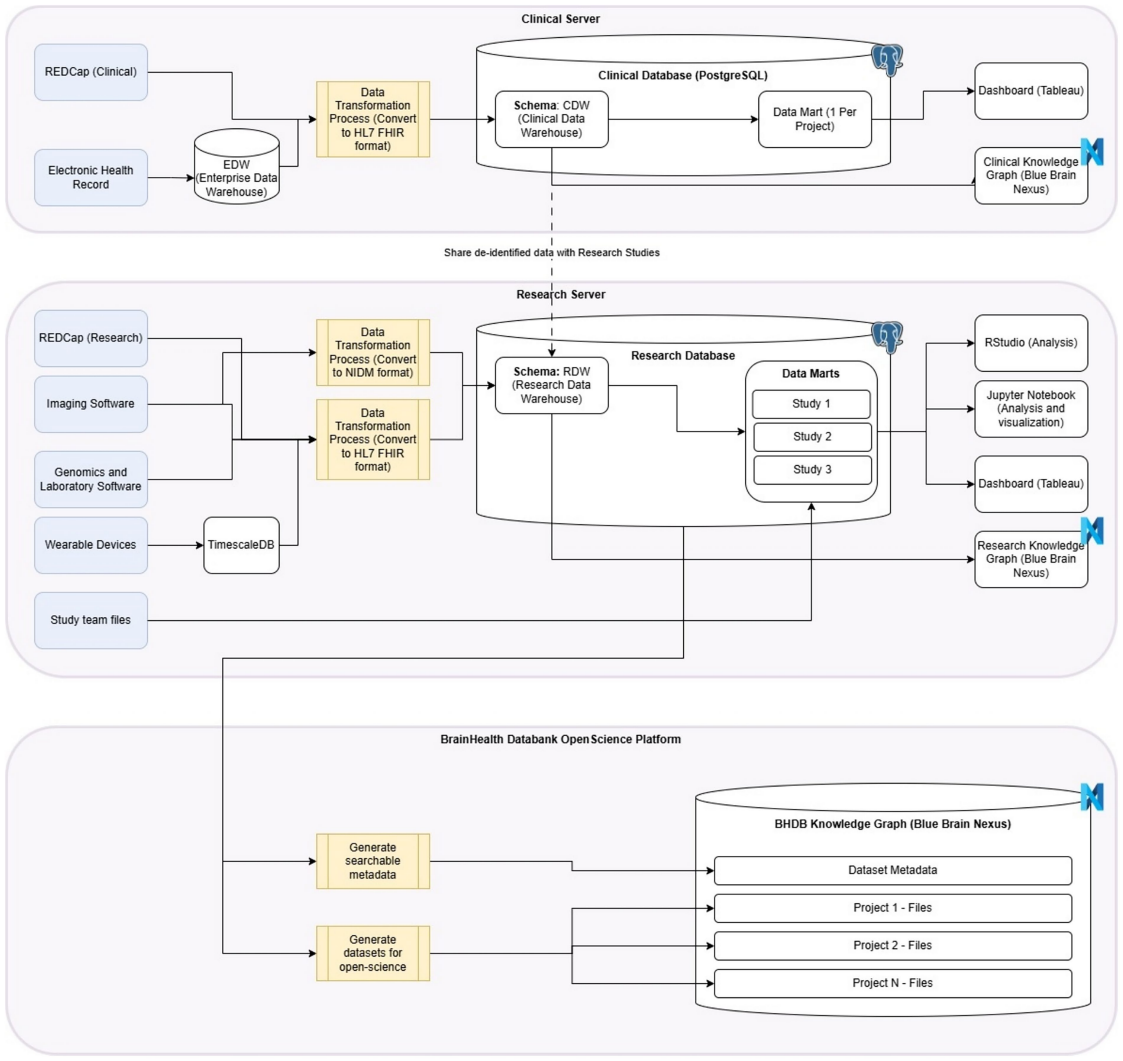
BHDB Data is collected through either the Clinical Pathway (Digitization of Care Pathways) or the Research Pathway (Integration of Research Methods into Care Pathways). After it is transformed (e.g., to HL7 FHIR format), data is stored in either a Clinical Data Warehouse (CDW) or Research Data Warehouse (RDW) in Postgres data marts, where it can then be stored in a Blue Brain Nexus Knowledge Graph, as well as analyzed and visualized through data system tools (AI and Data Driven Personalized Care). Data in the CDW can be transferred to the RDW and data can also be transferred from the RDW to the BrainHealth Databank Open Science Platform Blue Brain Nexus. This is where it can be accessed by the BHDB Portal for data sharing (Open Science for Discovery and Innovation).

The infrastructure and processes that have been described here are connected from data collection (both clinical and research) to its end use (e.g., analysis, data sharing), creating a seamless data flow for the entire BHDB. This data pathway connects every aspect of the BHDB (i.e., Digitization, of Clinical Care Pathways, Integration of Research Methods into Care Pathways, Artificial Intelligence and Data-Driven Personalized Care, Open Science for Discovery and Innovation). The technical data flow is described in a diagram (Figure 2).

## Results

### Governance and implementation at CAMH

The successful implementation of the BHDB LMHS at the CAMH was supported by a comprehensive and inclusive governance framework. This framework engaged a diverse range of partners, including clinicians, researchers, privacy and ethics experts, patient and family representatives, and data scientists. The governance structure, including the Steering Committee, the Data and Biosample Access Committee, and the Biobank Working Group, ensured that the system was aligned with clinical needs, ethical standards, and best practices in data management.

The governance model facilitated continuous feedback, adaptation, and decision-making. Standard operating procedures (SOPs) for data lifecycle management were established to ensure consistency and transparency in data handling. Partner participation was extensive, with 83 partners attending 181 meetings held and 95% of partner feedback leading to actionable changes in the system on the basis of progress reports. Repeated use of the PPEET (Abelson et al., 2016) revealed that 100% of patients and family partners were satisfied with their level of involvement. Patients and family advisors coauthored an article describing coleadership with patients and family partners at the BHDB (Yu et al., 2023). As part of our commitment to transparency and to support broader adoption of our methods, the BHDB governance documents and SOPs are provided as part of this publication.

### Enhanced data quality and integration

**Figure 3:**
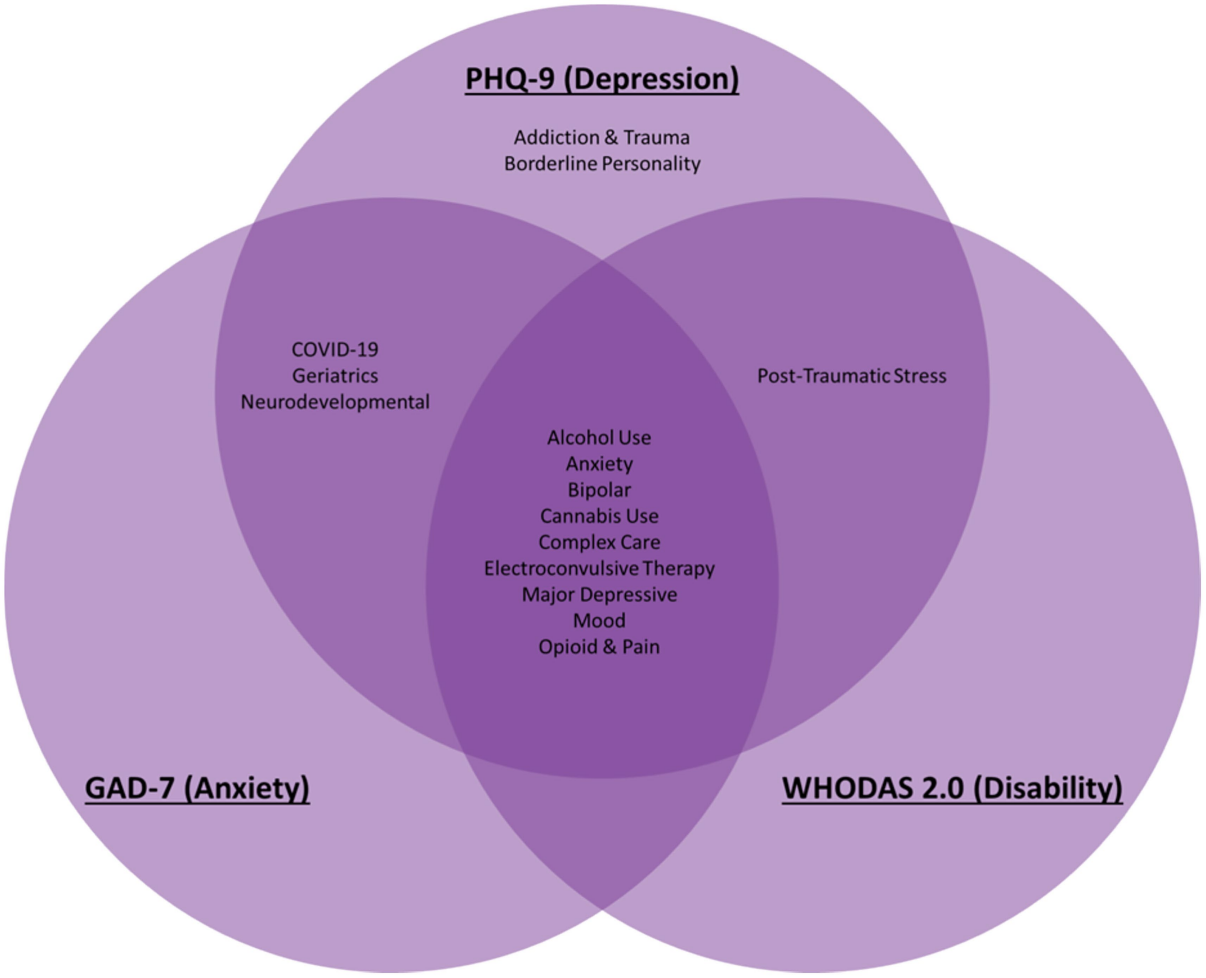
Common transdiagnostic data elements were assessed across diverse psychiatric disorders throughout all life stages in the BrainHealth Databank (BHDB) framework. The most commonly used standardized measures are presented here: the PHQ-9 for depression the GAD-7 for anxiety, and WHODAS 2.0 for disability. They are used across conditions such as alcohol use disorder (AUD), bipolar disorder (BD), borderline personality disorder (BPD), major depressive disorder (MDD), and treatment modalities such as electroconvulsive therapy (ECT), enabling personalized, data-driven clinical decision support.

The BHDB has successfully integrated data from 43 care pathways across 15 clinics at CAMH, encompassing 41 standardized questionnaires. Clinics, based on their patients’ needs, decide which standardized measures to use. The most used standardized questionnaires by clinics were the Patient Health Questionnaire-9 (PHQ-9) for depression, the General Anxiety Disorder 7 (GAD-7) for anxiety, and the World Health Organization Disability Assessment Schedule (WHODAS 2.0) for functional disability (Figure 3). This integration has facilitated detailed comparisons across patient groups and conditions, enhancing the personalization of care provided (Hawley et al., 2021).

As of November 1, 2024, the BHDB holds data on 35,434 patients, with 12,856,850 data points collected by 223 clinicians (52% of CAMH physicians). Standardized data collection protocols through REDCap, a secure web application for managing electronic surveys and patient self-assessments, resulted in 27% more patients with complete demographic and socioeconomic data than patients whose data were collected through clinical interviews, improving the completeness of the data available for research and clinical decision-making.

### Data integration, knowledge graphs, and technology stacks

Central to the BHDB’s data architecture is the implementation of a robust knowledge graph, built via the Blue Brain Nexus (Sy et al., 2023), which integrates multidimensional data across clinical and research domains. The knowledge graph serves as a centralized repository that links diverse datasets from the EHR and from research projects, including clinical assessments, patient-reported outcomes, medication histories, genetic data, and biobank samples. The integration of these datasets is facilitated by the use of the FHIR (Bender and Sartipi, 2013) standard as the common data model, ensuring that all the data are harmonized and interoperable across different systems.

The Blue Brain Nexus acts as the data management backbone, supporting the ingestion, storage, and querying of the data (Sy et al., 2023). Incoming data are converted to the FHIR format (Bender and Sartipi, 2013), which provides a standardized framework for representing health information. This standardization allows seamless data exchange and interoperability across platforms, making the data accessible and actionable for clinical decision-making and research purposes. The knowledge graph is continuously updated, with data becoming available in the knowledge graph the day after it is collected. This enables the BHDB to incorporate new data types and sources as the system expands.

### Biobank integration

The Biobank, managed by the Biobank Working Group, plays a crucial role in the BHDB by providing a rich repository of 8,146 biological samples collected from 54 studies that are linked to clinical and research data within the knowledge graph. The biobank supports the collection, storage, and use of biological samples, such as blood and tissue, which are essential for genomics and other biomarker studies. The integration of biological data with clinical and patient-reported outcomes within the knowledge graph enables comprehensive analyses that can identify molecular biology contributors to mental health conditions, ultimately informing personalized treatment strategies. The support of the CAMH Biobank and Molecular Core Facility has been acknowledged in 18 peer-reviewed publications since its inception in 2019, as determined by a literature search for the CAMH Biobank and Molecular Core.

### Development of data-driven clinical decision support

**Figure 4:**
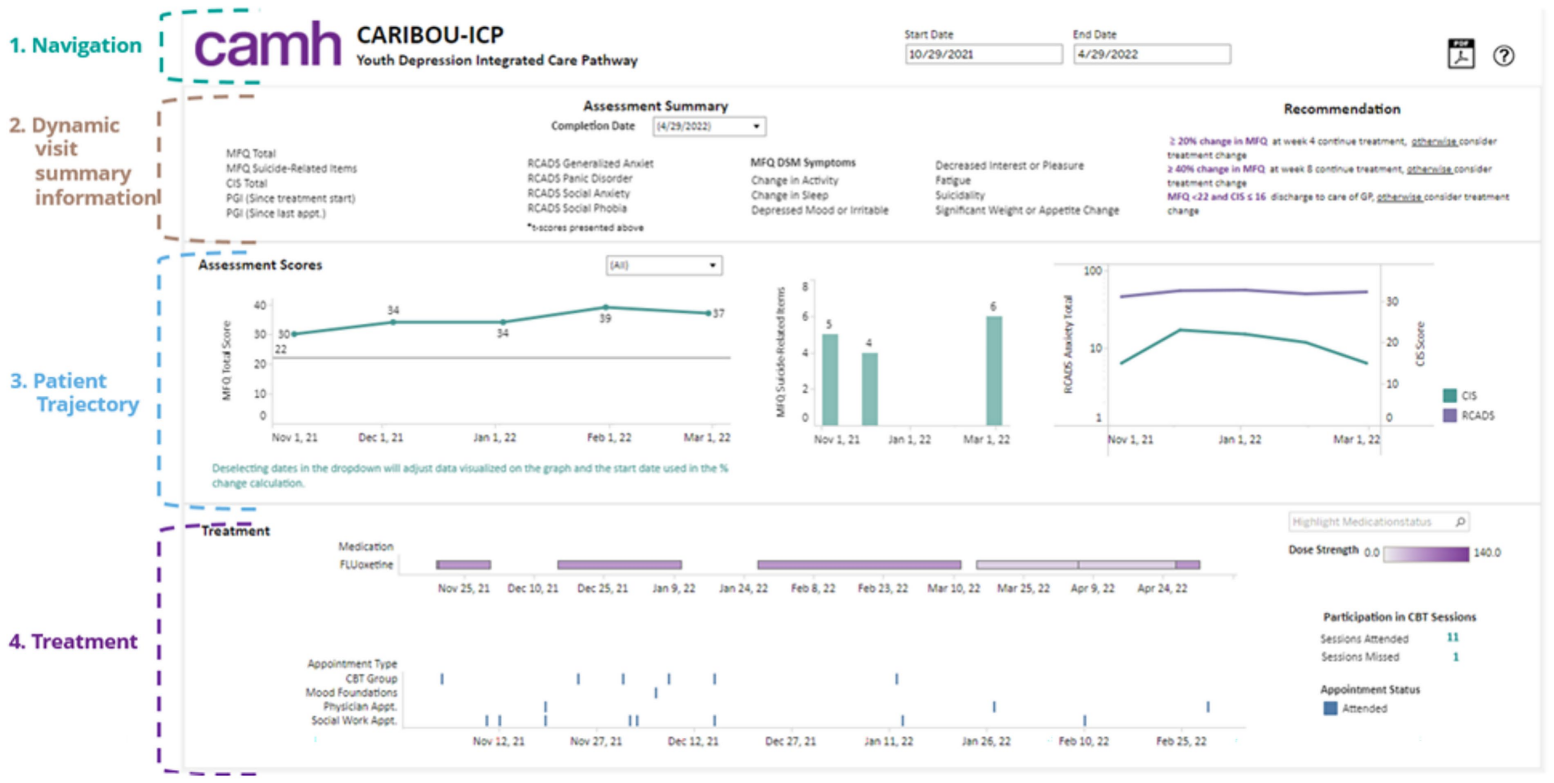
The CARIBOU-ICP dashboard illustrates the comprehensive tracking of a youth patient’s depression treatment within the CAMH Youth Depression Integrated Care Pathway (ICP). Key sections include (1) Navigation for managing timelines and appointments; (2) dynamic visit summaries displaying detailed assessment information for mood (MFQ), anxiety (RCADS), and functional impairment (CIS); (3) patient trajectories showing trends in key depression and anxiety scores over time; and (4) treatment details, including medication (fluoxetine) dosage and participation in cognitive behavioral therapy (CBT) sessions, which help to inform personalized clinical recommendations.

The BHDB developed and deployed five clinical decision support dashboards that leverage the knowledge graph to provide clinicians with comprehensive and contextually relevant insights into data along patients’ treatment journeys, including medical history, current medications, potential side effects, risk factors, and treatment progress. Figure 4 shows the dashboard developed for a care pathway for adolescents with depression, the CARIBOU-ICP (Courtney et al., 2020). These dashboards draw on the integrated data within the knowledge graph to seamlessly present a holistic view that highlights changes over time within the patient’s EHR.

The key features of decision support dashboards include the following:

- Patient trajectory visualization: Clinicians can visualize the patient’s journey through care, including key events such as diagnoses, medication changes, and clinical assessments. This helps in understanding the patient’s history and current status quickly.
- Treatment Management: The system provides detailed information about current and past medications, including dosages, duration, and adherence, as well as clinician appointments (e.g., psychotherapy). Potential drug interactions and side effects are flagged, drawing from real-time data within the knowledge graph.
- Risk factor analysis: The dashboards highlight risk factors that may affect treatment outcomes, dynamically updating these factors on the basis of ongoing data collection.
- Side Effect Monitoring: Clinicians receive alerts about emerging side effects associated with treatments, generated by analyzing patterns within the knowledge graph.
- Treatment progress and outcomes: real-time tracking of treatment responses allows clinicians to adjust care plans on the basis of standardized measures, ensuring that treatment aligns with the patient’s evolving needs.

These dashboards are codeveloped with clinicians to be user friendly and seamlessly integrated with the EHR, ensuring that the wealth of data available in the knowledge graph can be easily interpreted and applied in clinical practice. Clinicians reported positively on the usability of the dashboard and how they were able to integrate it into team meetings (Hassan et al., 2024b). The potential impact of these tools on clinical outcomes has been observed, such as a further 6 percentage point reduction in symptom severity (as measured by the Moods and Feelings Questionnaire [MFQ]) and an increase of 2 percentage points in functional scores (as measured by the Columbia Impairment Scale [CIS]) by the time of discharge after the implementation of these tools.

In addition, these dashboards will integrate machine learning predictive models developed through the AI & Data-Driven Personalized Care component. An example of a model currently under development is one that is trained on clinical data from the MDD-ICP to predict if a patient will discontinue treatment before discharge. The goal is to provide clinicians with a notification in the dashboard to encourage them to identify and potentially mitigate the risk of treatment discontinuation.

### Increased adoption and clinical utilization

The deployment of digital tools within the BHDB, including the five decision support dashboards, REDCap, and the MyCAMH Portal (a web portal for patients to access their own data), facilitated an increase in the adoption of MBC practices. Specifically, there has been a fourfold increase (from 5 to 20 clinicians within the MDD-ICP) in clinician adoption of MBC workflows and a threefold increase (from 12 to 49 patients per month in the MDD-ICP) in patient enrollment in MBC (Hawley et al., 2021). These tools have been well received by users, with 94% of clinicians reporting that they find the tools useful in their daily practice. Clinician engagement has reached 70% of all CAMH clinicians, as determined by the number of clinicians who use the dashboard at least five times greater than the number of CAMH physicians. This high level of satisfaction and adoption suggests that the BHDB may be a key contributor to promoting data-driven clinical practices and improving patient care.

### Integration of research and clinical practice

**Figure 5:**
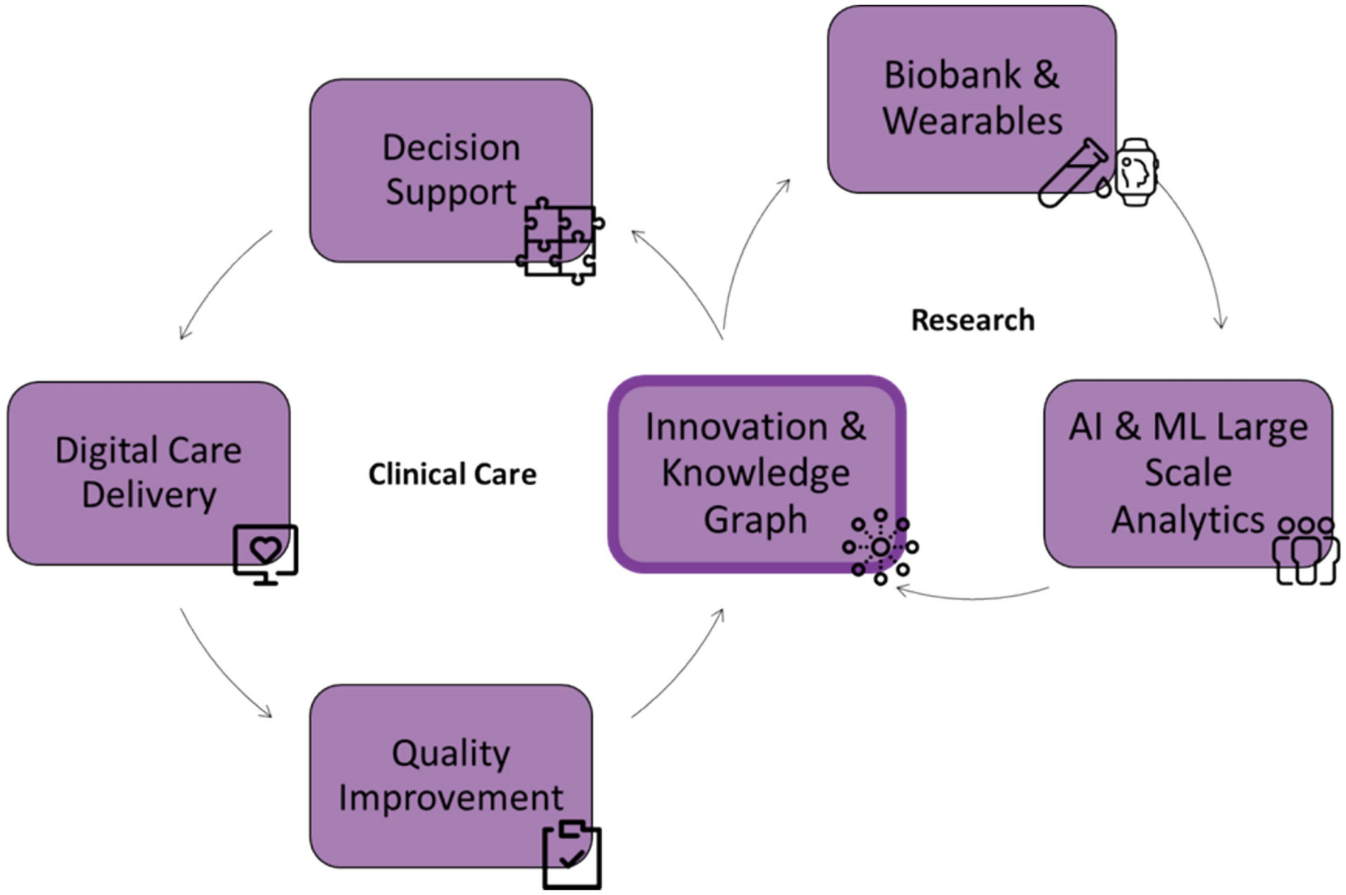
The integrated care and research framework of the BrainHealth Databank (BHDB) illustrates a continuous feedback loop between clinical care and research. On the clinical care side, digitized decision support and care delivery via electronic health records (EHR) support quality improvement, driving innovation. The research side includes biobanks and wearable data, and AI & machine learning (ML) analytics, which generate insights from large-scale data to drive innovation. Central to this framework is a knowledge graph that connects and integrates clinical and research data, fostering continuous innovation and improving outcomes across both domains.

A core achievement of the BHDB is its ability to bridge the gap between research and clinical practice. By incorporating research measures into clinical pathways, the BHDB facilitates continuous learning and innovation within a Learning Health System (LHS) framework (Friedman and Macy, 2014) (Figure 5). For example, the ongoing study within the major depressive disorder integrated care pathway (MDD-ICP) leverages data on sleep patterns to assess and refine treatment strategies. Clinicians gained insight into the relationship between circadian rhythm and inconsistent activity patterns and the symptoms of their patients from ongoing analysis of the continuously collected data (conference abstract in press by Verma et al.). The BHDB has supported 8 studies to date, with an average of 463 participants per study. This research has resulted in 6 peer-reviewed publications (Cleverley et al., 2024; Quilty et al., 2024; Dickie et al., 2024; Agarwal et al., 2023) and conference presentations (unpublished). This integration demonstrates the capacity of the BHDB to advance evidence-based care by directly linking research findings with clinical applications.

### Patient and family partners engagement

Patient and family engagement has been a central focus of the BHDB operating on a partnership model with shared decision-making, such as membership in the BHDB Steering Committee (Yu et al., 2023). The BHDB applied an evolving, dynamic engagement strategy throughout the development of the MyCAMH portal and patient journey dashboards. These tools were codesigned with patient advisors to ensure that the data presented would be meaningful to patients (Yu et al., 2023). They are used by a significant number of patients to access and interact with their MBC data. The MyCAMH portal recently started registering patients. There are currently 198 unique and active patient accounts. The MyCAMH portal and the patient journey dashboard are potential drivers for patient adoption and engagement with MBC. Continuous iteration of these tools, driven by patient feedback, will ensure that they remain relevant and effective in supporting patient-centered care.

### Beyond CAMH

The framework used to develop the BHDB has already had an impact beyond CAMH. The governance structure and technical infrastructure of the CAMH are currently being implemented in 13 organizations, such as hospitals and research institutes, across Canada. For example, the BHDB is currently being used as the foundation for platforms supporting data collection, analysis, visualization, storage, and within-network sharing. These platforms are the Cardio-Neuro-Mind Data Platform, which is a network of researchers focused on cardiological, neurological, and psychiatric disorders from hospitals in Ottawa and the Child and Youth Mental Health Insight Platform (Hill et al., 2022), which is a partnership of youth clinics. In addition, peer-reviewed BHDB governance manuscripts and study protocols have 53 collective citations.

### Comparison with related data repositories for secondary research

The environmental scan of related secondary research data repositories found thirteen data repositories providing data access for unspecified secondary use with accessible policies, procedures, or consent forms. The results of this scan are summarized in Table 1.

**Table 1:** Environmental scan of related data repositories for secondary research.

Project/platform	Country	Data type(s)	Access type(s)	Access eligibility	Accessing data
CanPath	Canada	Human subjects research data, and blood and urine samples	Controlled Access	Research Ethics Board approval and CanPath Access Office, Scientific Director(s), and Access Committee approvals	Access to data and physical samples (no further information provided)
Canadian Consortium on Neurodegeneration in Aging (CCNA)/ LORIS/ COMPASS-ND	Canada	Human subjects with neurodegeneration and dementia research data and tissue samples	Controlled Access	CCNA Investigator and Data Access Request Form or non-CCNA Investigator with background materials and project outline approved by Publications and Data and Biological Sample Access Committee	Access to data on LORIS; Access to physical samples
Ontario Brain Institute (OBI) Brain-CODE	Canada	Human subjects neuroscience research data	Controlled and Open Access	Data Access Request and Committee Review approval	Access in secure virtual analytics workspace, but where this is not feasible, local download access can be requested
NIMH Data Archive (NDA)	US	Human subjects research data	Controlled and Open Access	Research need, Principal Investigator eRA Commons Account, Institutional Association, Active Federal Wide Assurance, NDA Account; Submit a Data Access Request with review by NIH-Staffed Data Access Committee	Use Download Manager to create a data package via the NDA Query Tool; Download to users computer
BioVU Vanderbilt	US	Patient blood samples	Controlled Access	Vanderbilt faculty member, Institutional Review Board and Scientific Review Committee approval, Data Use Agreement	Access to physical samples (no further information provided)
Mass General Brigham Biobank	US	Patient data and blood samples	Controlled Access	Ethics board approval for researchers and staff	Access to data and physical samples (no further information provided)
All of Us Research Hub	US	Patient data, and blood, saliva, and urine samples	Controlled Access	Meet data security standards, ethics training through their program, Code of Conduct, All of Us Institutional Review Board approval	Access on a secure cloud-based environment; Access to physical samples
ABCD Study	US	Adolescent human subjects research data, and blood, saliva, urine, and hair samples	Controlled Access	Eligible researchers with a valid research use at a research institution with Federal Wide Assurance	Access to data only (not samples; no further information provided)
Center for Data Driven Discovery in Biomedicine (D3b) -Children’s Hospital of Philadelphia (CHOP)	US	Pediatric Patient data and bio specimens	Controlled and Open Access	Varies by subproject; Involves appropriate affiliations, data security best practices, ethics board approval, request form, or Access/Scientific Committee approval	Varies by subproject; Access to data and physical samples
OpenNeuro	US	Human subjects imaging research data	Open Access	N/A	Download
Dementias Platform UK (DPUK)	UK	Human subjects with dementia research data	Controlled Access	Affiliated with research organization, application form, Data Guardian review and approval, Data Access Agreement	Access in analysis platform (cannot be downloaded)
UK Biobank	UK	Human subjects research data, and blood, saliva, and urine samples	Controlled Access	Relevant scientific and ethics approvals including Board of Directors and Access Committee approval, data use agreement, required to publish results	Access data on a cloud-based platform, or download to users computer; Access to physical samples
Radboud Data Repository	Netherlands	Research data	Open and Controlled Access	Varies by data access level; Involves Data Use Agreement, access request, Collection Manager review and approval	Download

## Discussion

The implementation of the BHDB LMHS at the CAMH has demonstrated substantial progress in advancing mental health research and care. However, several challenges and considerations remain critical to ensuring the long-term success and sustainability of the initiative. This discussion explores the key challenges, the strategies employed to overcome them, and the broader implications of the BHDB for mental health care.

### Ensuring high-quality data

Maintaining high data quality is paramount for the success of any data-driven health system, particularly one as ambitious as the BHDB. The accuracy, completeness, and consistency of the data collected directly influence the reliability of clinical decision-making and research findings (Lipworth, 2019). Despite the BHDB’s standardized data collection protocols, challenges such as missing data due to missed appointments or incomplete assessments remain. These gaps can significantly affect the validity of research outcomes. The BHDB has addressed this issue by closely collaborating with clinicians to map complete MBC pathways from intake to discharge. The development and implementation of digital support tools have been instrumental in promoting clarity and reducing the quantity of missing data. The BHDB adopted the Canadian Institute for Health Information’s Data Quality Framework (CIHI, 2017). Continuous monitoring and iterative improvements to these protocols are essential for maintaining high standards of data quality as the system evolves. The BHDB is currently working on reducing data entry errors, improving data consistency across pathways, and optimizing the average time from data entry to availability.

### Achieving interoperability across systems

Interoperability remains a significant challenge for large-scale data integration efforts such as the BHDB. The integration of data from diverse systems and institutions is complex and often hindered by differences in data standards, formats, and technologies (Barch et al., 2016). These issues can obstruct seamless data sharing and integration, particularly when a patient or research participant is treated in multiple clinics or involved in various studies. A critical aspect of achieving high data quality and interoperability across diverse systems has been collaboration with the CAMH Standards and Measures Committee. This committee reviews the usage of measures proposed by clinicians to ensure the validity and consistency of the measures used at CAMH. This partnership has facilitated the harmonization of data formats and the promotion of common data elements across different care pathways. By increasing standardization, the BHDB has ensured that data are comparable and easily shared across institutions, enhancing its utility for both clinical and research purposes (Barch et al., 2016).

### Protecting patient privacy

The continuous collection of data throughout a patient’s care journey, while invaluable for improving outcomes, raises significant concerns about privacy and data security (van Staa et al., 2016). Ensuring compliance with privacy regulations and maintaining patient trust are critical for BHDB success. Patients must trust that their data will be protected so that the BHDB can collect accurate data that can be trusted by clinicians. The BHDB has implemented robust data governance and security measures, including deidentification processes, limiting personal identifying information, and conducting risk-of-reidentification analyses. These measures are designed to protect patient privacy while maximizing the utility of the data collected. However, the dynamic nature of data collection and the increasing sophistication of AI tools necessitate ongoing vigilance and adaptation of privacy measures to mitigate new risks as they emerge (Jacobs et al., 2021).

### Engaging a wide range of partners

The success of the BHDB is deeply rooted in its inclusive approach to partner engagement. Engaging clinicians, researchers, private and ethical experts, patients, and family representatives in decision-making processes has been essential in ensuring that the system meets the diverse needs of all partners. However, balancing these diverse needs and priorities can be challenging, requiring continuous effort and effective communication. The BHDB governance structure, which includes the steering committee and patient and family engagement team, has been instrumental in ensuring timely and sufficient input from all partners. Codesigning tools with clinicians and patients ensures that the digital tools developed are user friendly, relevant, and aligned with clinical practice, thereby enhancing partner trust and system adoption (Yu et al., 2023).

### Adoption of new technologies

The adoption of new digital tools and technologies, while necessary for advancing mental health care, can lead to resistance from clinicians and other users due to potential negative impacts from their implementation, such as burnout (Jankovic and Chen, 2020). Ensuring that these tools are seamlessly integrated into existing workflows and are user friendly is crucial for their acceptance and effective use. Similarly, building trust through robust data governance and secure data protection, as well as continuous evaluation and following codesign principles, is required for user acceptance (Hassan et al., 2024a). The BHDB has provided comprehensive training and support to encourage the adoption of new technologies. Continuous data collection allows for the ongoing assessment of the impact of these tools. Iterative improvements based on user feedback have been built into the system, ensuring that the digital tools evolve in response to the needs of the clinicians and patients who use them (Hassan et al., 2024b). A novel element of our approach is the integration with CAMH’s adoption-centric AI governance, driven by evidence-based frameworks. Iterative evaluation will be embedded in the end-to-end process, from model development through to implementation and sustainability (Hassan et al., 2025). This ensures there is robust governance for the end-uses of data from the BHDB.

### Scaling the initiative

Expanding the BHDB to additional hospitals and health systems presents significant logistical and operational challenges. Ensuring consistent data collection practices, maintaining data quality, and achieving interoperability across different institutions are critical for successful scaling (Gaveikaite et al., 2018; Allen et al., 2021; Enticott et al., 2021). Each new environment may have unique requirements, making scalability a complex and nuanced process (Enticott et al., 2021). The BHDB was designed with scalability in mind, facilitating the integration of research methods into more care pathways, applying machine learning to a broader patient population, and increasing data sharing and reuse through open science principles (Poupon et al., 2017). The Cardio-Neuro-Mind Data Platform and Child and Youth Mental Health Insight Platform instances of the BHDB function with the same underlying technology as the BrainHealth Databank but have differences in data governance, demonstrating that the underlying processes can adapt to the needs of different populations. In addition, the data across all of these platforms is standardized with FHIR ontology (Bender and Sartipi, 2013) allowing for federated analyses (provided all data governance requirements are met). This creates the possibility of large sample size studies with more heterogenous participants. From these experiences, the lessons learned were that future efforts to scale the BHDB require close collaboration with partner institutions to address these challenges and adapt the system to diverse clinical settings.

### Robust governance structures

The BHDB’s comprehensive governance framework has been essential in addressing challenges related to data quality, privacy, and partner engagement. Governance structures such as the Data and Biosample Access Committee ensure that data requests are managed ethically and that privacy considerations are met. Standard operating procedures (SOPs) for data lifecycle management have been established to ensure consistency and transparency in data handling. Continuous feedback and communication between working groups and the Steering Committee have supported timely input and decision-making, ensuring that the system remains responsive to the evolving needs of its users.

### Continuous feedback and adaptation

A key strength of BHDB is its ability to adapt on the basis of real-world evidence and user input. Continuous feedback mechanisms have been implemented to refine tools and processes, ensuring that the system remains effective and relevant to different health systems. Involving front-line clinicians and hospital administrators in the design and implementation of digital support tools has been particularly effective in reducing the quantity of missing data and improving data clarity. This adaptability is crucial for addressing the dynamic needs of mental health care and ensuring that the BHDB continues to deliver high-quality care and support innovative research.

### Comparison with related data repositories for secondary research

The BHDB is hosted in Canada, which is a minority of the sample. This benefits Canadian researchers that for ethical reasons require data that will comply with Canadian privacy legislation. The BHDB shares only institutional human subjects’ research data (i.e., from CAMH research studies only), which aligns with the majority of other repositories. This provides a more limited sample than some multi-site study or population-based repositories in the sample. The BHDB shares both research data and biosamples, which is in alignment with the majority of repositories that share data or both data and biosamples. However, the BHDB only shares biosamples with institution-affiliated researchers (i.e., not external researchers without a CAMH collaborator). The BHDB provides controlled-access to data, which aligns with most other repositories. Access requirements are the BHDB Data and Biosample Access Committee review and ethics approval, as well as a Data Use Agreement for external requestors, which are similar requirements that other repositories have. The BHDB will allow access to data via direct download to the user’s computer, which is less common than secure platform access in the sample. In addition to this process, CAMH researchers can request access to clinical BHDB data after obtaining the necessary ethical approvals. For example, comparing the BHDB with the UK Biobank, there are areas of alignment (e.g., robust governance, controlled-access with similar controls) but also some differences (e.g., the BHDB is smaller and focused on only one institution, sharing biosamples only with CAMH researchers, while the UK Biobank biological samples with external researchers worldwide).

### Caveats and limitations

While the BHDB has made significant strides, several limitations and caveats must be acknowledged. Data quality issues persist, particularly with regard to missing data and inconsistencies in data collection methods. Despite efforts to standardize data collection, variations in how assessments are conducted can introduce biases and affect the reliability of research findings. Privacy concerns also remain a critical issue, as the continuous collection of sensitive personal health information requires ongoing vigilance to ensure compliance with regulations and maintain patient trust (van Staa et al., 2016; Jacobs et al., 2021). Patient and family partner engagement, while a strength of the BHDB, can also present challenges in balancing the diverse needs and priorities of different groups (Yu et al., 2023). Ensuring timely and sufficient input from all partners requires continuous effort and effective communication. Additionally, the adoption of new digital tools and technologies continues to face resistance from some clinicians, highlighting the need for continuous training and support (Hassan et al., 2024b). Finally, scaling the BHDB to other institutions presents logistical and operational challenges that need to be carefully managed to maintain data quality and achieve interoperability across institutions.

### Future directions

Looking ahead, the BHDB is poised for expansion, with the potential to extend its successful model to other institutions, thereby accelerating research and linking mental health to physical health conditions. This expansion will involve establishing collaborative partnerships, ensuring interoperability, and maintaining high standards of data security and ethical compliance. A key strategy will be the application of open science principles (Poupon et al., 2017) through the BHDB portal, which will promote widespread collaboration and accelerate the pace of discovery and innovation in mental health care. Additionally, by openly sharing all documentation, consent models, and standard operating procedures (SOPs), the BHDB aims to support transparency and facilitate the broader adoption of its methods across the healthcare landscape.

## Conclusion

The BHDB provides a robust example for integrating clinical practice, research, and data analytics within an LHS framework. Its comprehensive and integrated dataset supports evidence-based, personalized care, enhances clinical decision-making, and drives innovation. As the BHDB continues to grow, it will play a critical role in improving patient outcomes and advancing our understanding of mental health conditions, ultimately contributing to a more effective and responsive healthcare system.

## Data Availability

The original contributions presented in the study are included in the article/supplementary material, further inquiries can be directed to the corresponding author.
